# Evolutionary responses to crude oil from the Deepwater Horizon oil spill by the copepod *Eurytemora affinis*

**DOI:** 10.1111/eva.12502

**Published:** 2017-08-16

**Authors:** Carol Eunmi Lee, Jane Louise Remfert, Taylor Opgenorth, Kristin M. Lee, Elizabeth Stanford, Joseph William Connolly, Jinwoo Kim, Sarah Tomke

**Affiliations:** ^1^ Center of Rapid Evolution (CORE) and Department of Integrative Biology University of Wisconsin, Madison Madison WI USA

**Keywords:** adaptation, Macondo Prospect, pollution, polycyclic aromatic hydrocarbons, quantitative genetics, toxicity, WAF, water‐soluble fraction, zooplankton

## Abstract

The BP Deepwater Horizon Oil Disaster was the most catastrophic offshore oil spill in U.S. history, yet we still have a poor understanding of how organisms could evolve in response to the toxic effects of crude oil. This study offers a rare analysis of how fitness‐related traits could evolve rapidly in response to crude oil toxicity. We examined evolutionary responses of populations of the common copepod *Eurytemora affinis* residing in the Gulf of Mexico, by comparing crude oil tolerance of populations collected before versus after the Deepwater Horizon oil spill of 2010. In addition, we imposed laboratory selection for crude oil tolerance for ~8 generations, using an *E. affinis* population collected from before the oil spill. We found evolutionary increases in crude oil tolerance in the wild population following the oil spill, relative to the population collected before the oil spill. The post‐oil spill population showed increased survival and rapid development time in the presence of crude oil. In contrast, evolutionary responses following laboratory selection were less clear; though, development time from metamorphosis to adult in the presence of crude oil did become more rapid after selection. We did find that the wild population, used in both experiments, harbored significant genetic variation in crude oil tolerance, upon which selection could act. Thus, our study indicated that crude oil tolerance could evolve, but perhaps not on the relatively short time scale of the laboratory selection experiment. This study contributes novel insights into evolutionary responses to crude oil, in directly examining fitness‐related traits before and after an oil spill, and in observing evolutionary responses following laboratory selection.

## 1 INTRODUCTION

The BP Deepwater Horizon Oil Disaster of 2010 was the most catastrophic offshore spill in U.S. history, with an estimated 4.6 million barrels of oil spilling into the Gulf of Mexico over 86 days (Griffiths, 2012). Following this oil spill, 1.6–2.6 × 10^4^ metric tons of hydrocarbons accumulated on the seafloor and persisted long after oil was no longer present in the water column (Montagna, Baguley, Cooksey, & Hyland, 2017; Yan et al., 2016). Carbon isotope tracers revealed that transformed oil products persisted for years, particularly in low energy environments (Pendergraft & Rosenheim, 2014). Additionally, the labile hydrocarbons were transferred to the planktonic food web, particularly to mesozooplankton, consisting mostly of calanoid copepods (Graham et al., 2010).

While the adverse effects of the Deepwater Horizon oil spill endure to the present day, relatively little is known regarding the potential evolutionary responses of organisms to the toxic effects of crude oil. Such information is crucial, as evolutionary responses are critically important in cases where acute stress exceeds the capacity of organisms to respond through phenotypic plasticity. Additionally, many species that dominate the water column, such as mesozooplankton (e.g., copepods), are weak swimmers and are deficient in their ability to disperse. In addition, brackishwater species in the Gulf would likely have limited dispersal capacities due to their restricted salinity tolerances (see below). Given the low dispersal capacity of many marine and brackish organisms, studying their evolutionary responses to crude oil toxicity is an essential component toward understanding the potential for recovery of populations from oil spill disasters.

Crude oil is a complex mixture containing many unidentified and toxic compounds (Liu & Kujawinski, 2015; Melbye et al., 2009; Robson, Sutton, McCormack, Chilcott, & Rowland, 2017). Within crude oil, components that are particularly toxic due to their bioavailability are contained within the “water‐accommodated fraction” (WAF), consisting of the “water‐soluble fraction” (WSF) and microscopic oil droplets (Liu & Kujawinski, 2015; Melbye et al., 2009; Neff, 2002). The water‐soluble fraction (WSF) is a solution of low molecular mass hydrocarbons naturally released from petroleum hydrocarbon mixtures in contact with water (Liu & Kujawinski, 2015). The toxic constituents of the water‐soluble fraction (WSF) include polar fractions containing many cyclic and aromatic sulfoxide compounds, as well as polycyclic aromatic hydrocarbons (PAHs), such as naphthalene (NAPH) and dimethylnaphthalene (C2‐NAPH) (Melbye et al., 2009). The PAHs have been found to be quite toxic for many organisms, including copepods (Almeda et al., 2013; Barata, Calbet, Saiz, Ortiz, & Bayona, 2005; Bejarano, Chandler, He, & Coull, 2006; Berdugo, Harris, & O'Hara, 1977; Cohen, McCormick, & Burkhardt, 2014; Ott, Harris, & O'Hara, 1978; Saiz, Movilla, Yebra, Barata, & Calbet, 2009).

The few studies that have revealed rapid evolutionary responses of organisms to the toxic effects of crude oil have focused predominantly on microorganisms. For instance, laboratory selection experiments revealed that freshwater and marine phytoplankton collected from pristine uncontaminated sites are able to evolve resistance to crude oil toxicity, presumably through selection on novel mutations (Carrera‐Martinez, Mateos‐Sanz, Lopez‐Rodas, & Costas, 2011; Romero‐Lopez, Lopez‐Rodas, & Costas, 2012). In bacteria, genes that encode alkane hydroxylases (AHs), responsible for crude oil degradation, are relatively widespread and could potentially be transferred to neighboring bacteria through horizontal gene transfer (Nie et al., 2014).

However, for larger multicellular organisms, adaptation to oil pollution might be far more difficult to achieve, given their longer generation times, lower rates of mutation, lower effective population size, and extremely rare incidence of horizontal gene transfer. Evolutionary adaptation to crude oil toxicity in animals would most likely need to rely on selection on standing genetic variation for crude oil tolerance within the populations. In an older study, imposing crude oil exposure for 2–10 days in marine gastropods revealed allele frequency shifts at two allozyme loci, suggesting an evolutionary response to crude oil, though the functional implications of these allele frequency shifts are unclear (Nevo & Lavie, 1989). In a comparison between populations of the Trinidad guppy *Poecilia reticulate* in oil‐polluted versus unpolluted streams, populations in oil‐polluted habitats showed little evidence for adaptation to oil pollution, but were actually maladapted to their local environment (Rolshausen et al., 2015). An intriguing comparative study found that the Gulf killifish *Fundulus grandis* at an oil‐contaminated site in the Gulf of Mexico exhibited changes in genome expression and larval CYP1A enzyme expression (a biomarker for PAH exposure) that was divergent from fish from uncontaminated sites, 1–4 months after the oil spill (Whitehead, Dubansky, et al. 2012). This study measured acclimatory (rather than evolutionary) responses of oiled versus non‐oiled controls, as it examined expression changes within the lifespan of the fish. A notable study comparing four pairs of pollution‐tolerant and intolerant populations of the killifish *Fundulus heteroclitus* found signatures of selection at genomic regions containing key genes involved in the aryl hydrocarbon receptor (AHR) signaling pathway, which mediates tolerance of PAHs (Reid et al., 2016).

These prior studies lead to the question on the extent and speed to which fitness‐related traits could evolve in response to crude oil toxicity in animals. Thus, the goal of this study was to test for rapid evolution of crude oil tolerance in the copepod *Eurytemora affinis*, a common resident in the Gulf of Mexico. Populations of *E. affinis* appeared to be largely absent from the Mississippi delta region of the Gulf of Mexico following the Deepwater Horizon oil spill, but returned to this area more than a year later (see section 2 for details). An extensive survey performed in 2010 found that the *E. affinis* habitats we sampled in the Gulf of Mexico had been exposed to high concentrations of total petroleum hydrocarbons (TPHs) and PAHs, in seawater, seafood, and sediment (Sammarco et al., 2013) (https://web.archive.org/web/20101126225809/http://leanweb.org:80/news/latest/testing-results-returning-with-high-levels.html). A rebound of *E. affinis* populations through dispersal is unlikely, given their limited long‐distance swimming capabilities. Moreover, salinity poses a major barrier to dispersal of *E. affinis* populations, which show heritable differences in their salinity tolerances (Lee, 1999, 2016; Lee & Petersen, 2003; Lee, Remfert, & Chang, 2007; Lee, Remfert, & Gelembiuk, 2003). This low dispersal is reflected in the highly genetically structured populations of *E. affinis*, even between proximate sites (Lee, 1999, 2000; Winkler, Dodson, & Lee, 2008). The critical question then is, how did populations of *E. affinis* rebound from the catastrophic effects of the Deepwater Horizon oil spill? Did they evolve physiological tolerance of crude oil toxicity?

To address the questions above, this study employed a common‐garden reaction norm approach to examine: (i) the presence of genetic variation in tolerance of crude oil toxicity in the populations, upon which natural selection could act, (ii) evolutionary responses following the oil spill in the wild, and finally (iii) evolutionary responses following selection in the laboratory. First, we determined whether the population of *E. affinis* in the Gulf of Mexico evolved elevated crude oil tolerance, by directly comparing populations collected before versus after the Deepwater Horizon oil spill, in terms of survival and development time at varying levels of crude oil exposure. Next, we attempted to induce selection in the laboratory for crude oil tolerance on the population of *E. affinis* collected before the Deepwater Horizon oil spill. We examined evolutionary responses of the populations to the “water‐accommodated fraction” of crude oil, which harbors the PAHs and other toxic compounds (Almeda et al., 2013; Faksness, Brandvik, & Sydnes, 2008; Forth, Mitchelmore, Morris, & Lipton, 2017; Liu & Kujawinski, 2015; Melbye et al., 2009).

Copepods, with their exceedingly large numbers and relatively short generation times (~15–20 days in *E. affinis*) are likely to experience significant evolutionary responses to environmental perturbations. In addition, the copepod *E. affinis* undergoes seasonal diapause on an annual basis, leaving a reservoir of resting eggs in the sediment (Ban & Minoda, 1992; Katajisto, 1996), which could contribute to standing genetic variation in the population. Following the oil spill, the eggs hatching from the sediment might have been subjected to natural selection in response to crude oil toxicity. In prior studies, *E. affinis* has been shown to rapidly evolve in response to environmental variables, such as temperature (Ketzner and Bradley 1982), salinity (Lee, Kiergaard, Gelembiuk, Eads, & Posavi, 2011; Lee et al., 2003, 2007), and food concentration (Lee et al., 2013).

Understanding evolutionary responses of organisms to crude oil toxicity could provide critical insights into the potential of populations to recover from oil spill disasters. This study is notable for applying a common‐garden approach to directly examine evolutionary responses before versus after an oil spill at a given location. Additionally, this study provides the first attempt to perform laboratory selection on animals in response to crude oil toxicity. Moreover, this study examined levels of standing genetic variation for crude oil tolerance within the pre‐oil spill population, as a measure of evolutionary potential.

The evolutionary analysis performed in this study has important ecological implications for the functioning of food webs in estuarine and coastal ecosystems. Copepods constitute the main grazers of algae and major food source for many important fisheries throughout the world. The copepod *Eurytemora affinis,* in particular, is a dominant member of coastal ecosystems throughout the Northern Hemisphere (Lee, 1999, 2000) and serves as the major food source for some of world's most important fisheries, such as herring, anchovy, salmon, and flounder (Kimmel, Miller, & Roman, 2006; Livdāne, Putnis, Rubene, Elferts, & Ikauniece, 2016; Shaheen et al., 2001; Viitasalo, Flinkman, & Viherluoto, 2001; Winkler, Dodson, Bertrand, Thivierge, & Vincent, 2003).

## 2 METHODS AND MATERIALS

### 2.1 Population sampling

In the Gulf of Mexico region, populations of the copepod *E. affinis* reside in the relatively protected waters of bayous, rather than in the open ocean. The two *Eurytemora affinis* populations (pre‐ and post‐oil spill) examined in this study were collected from the same location in the Gulf Mexico, Blue Hammock Bayou, LA (29 18′19.15″N, 91 7′42.48″W) proximate to Fourleague Bay, LA, USA. The “pre‐oil spill” population was collected using plankton tows, before the Deepwater Horizon oil spill of 2010 in March of 2006, at a salinity of 5 PSU (practical salinity unit [SI unit for salinity] ≈ parts per thousand salinity). On the other hand, the “post‐oil spill” population was collected after the Deepwater Horizon oil spill of 2010 in March of 2013 (see below).

Following the Deepwater Horizon oil spill of 2010, the copepod *E. affinis* appeared to be largely absent from its historical locations in the Mississippi delta region in the Gulf of Mexico. Typically, plankton tows in the bayous of the Mississippi River delta consistently yielded *E. affinis* in moderate abundance (pers. observation) (Vecchione, 1989). However, extensive sampling by the Lee lab in March of 2011 (~11 months after the Deepwater Horizon oil spill) revealed a marked absence of *E. affinis*, and the plankton community in general, in locations where they are typically common, including in Fourleague Bay, Blue Hammock Bayou (29°18′19.15″N, 91°7′42.48″W), and Oyster Bayou (29°08′36″N, 90°43′00″W), Louisiana, USA (G. Gelembiuk, Lee Lab, personal observation, March 16‐17, 2011). For Blue Hammock Bayou, where *E. affinis* is typically most abundant, plankton samples were taken at ten locations along the length of the bayou (~8 km), from the mouth at Four League Bay to the “narrows” at the eastern end of the bayou. 12–15 plankton tows were taken at each location, for a total of ~130 plankton tows throughout the bayou. Each plankton tow was taken for a duration of ~5 min against the flow of the current, from near the bottom (~7 m) to the top of the water column. Sampling took place from 9 a.m. to 5 p.m, for two days, on March 16‐17, 2011. This sampling effort around this time of year usually yields a few hundred to a few thousand individuals of *E. affinis*. However, in 2011 we found no *E. affinis* individuals in any of our samples.

Subsequently, sampling on March 24–25 of 2013 revealed the recovery of *E. affinis* populations, as well as other members of the plankton community at these locations. *E. affinis* was found at salinities ranging from 5 to 12 PSU. The “post‐oil spill” population used in this study was collected using plankton tows in Blue Hammock Bayou, LA at salinities 5.6–9 PSU, proximate to Oyster Bayou.

Both pre‐ and post‐oil spill populations were cultured in large numbers in the laboratory prior to the experiments. The populations were cultured in 5 PSU water (made using 0.22 μm filtered Lake Michigan water plus Instant Ocean^®^) in an environmental chamber at 12°C with a 15L:9D light cycle. The copepods were fed the crytophyte alga *Rhodomonas salina* (lab‐cultured) in excess. The antibiotic Primaxin (20 mg/L) was added at each water change to reduce bacterial growth.

### 2.2 Water‐accommodated fraction (WAF) of crude oil used for the oil‐exposed treatments

Crude oil (50 L) from the Deepwater Horizon spill, from the Macondo Well in the Mississippi Canyon Block 252 well (“MC 252”), was obtained from BP in August, 2012. Standard methods were used to produce the water‐accommodated fraction (WAF) of crude oil (Bejarano et al., 2006; Girling, 1989; Singer et al., 2001; Tsvetnenko & Evans, 2002). The WAF was made by making a 10:1 mixture of 5 PSU water to Macondo oil (3,600 mL of 5 PSU water + 400 ml of oil). The 5 PSU water was made from 0.2 μm filtered water from Lake Michigan with Instant Ocean^®^ added to achieve 5 PSU. The 10:1 mixture was gently mixed in an aspiration bottle for 22 hr under complete dark conditions. The newly formed WAF was pulled from the bottom of the aspiration bottle without disturbing the oil on the top and was stored in a loosely covered container at 12°C. See Almeda et al. (2013) and Cohen et al. (2014) for the composition of PAHs contained within the WAF of Deepwater Horizon Macondo Well oil.

Concentrations of WAF used in the experiments below were based on results from a preliminary common‐garden experiment, which measured the survival of ten *E. affinis* egg clutches split across five oil concentrations, namely 0 (control), 25%, 50%, 75%, and 100% WAF. At these oil concentrations, survival from hatching to metamorphosis was 92.3% ± 0.3 (control, 0% WAF), 91.3% ± 0.3 (25% WAF), 86.4% ± 0.4 (50% WAF), 68.4% ± 0.5 (75% WAF), and 33.3% ± 0.5 (100% WAF). Based on these results, 75% (72.5% in the second experiment) and 100% WAF were chosen as the treatment oil concentrations, as they showed substantial impacts on survival.

### 2.3 Common‐garden reaction norm experiment to compare response to crude oil of pre‐ and post‐oil spill populations

A common‐garden experiment was performed to compare responses to crude oil between the “pre‐oil spill” (collected before the oil spill) and “post‐oil spill” (collected after the oil spill) populations of *E. affinis* collected from the wild (see section 2.1 above). A common‐garden approach is used to assess the heritable genetic differences between populations by rearing and observing different populations under common conditions, and thus removing the effects of acclimation to different environments (i.e., removing the effects phenotypic plasticity). Thus, the difference in response observed between the populations under common‐garden conditions would represent the evolutionary differences between the populations, namely, the evolutionary changes in tolerance of crude oil from before the oil spill to after the oil spill.

Eight ovigerous females from each population were chosen at random and the eggs sacs were excised and split six ways across three WAF concentrations (0% [control], 75% and 100% WAF) with replicate vials for each treatment. Each full‐sibling clutch represented a distinct genotype. Between 10 to 12 eggs were split between two replicate vials per clutch for each oil concentration and population. With eight clutches per treatment, there were a total of 88–92 individual copepods per oil concentration for each population. Each 20 ml scintillation vial contained 10 ml of 5 PSU water. The vials were placed in racks and reared in environmental chambers at 12°C with a light cycle of 15L:9D and fed the alga *Rhodomonas salina* in excess. Approximately 75% of the water was removed and new water was added to each vial during weekly water changes. New batches of WAF mixtures were made within 24 hr prior to every water change.

Numbers of individuals at each life stage were counted every other day in the treatments, until death or reaching adulthood. Survival was analyzed as binary data (dead or alive), for hatching (hatched or not hatched for each individual egg), survival from hatching to metamorphosis (dead or alive for each individual nauplius), and survival from metamorphosis to adult (dead or alive for each individual copepodid). Development time was measured in terms of number of days from hatching to metamorphosis and number of days from metamorphosis to adult. Because developmental stage was recorded every other day, development time was averaged as the midpoint between observations. Metamorphosis was recorded at the transition from the life stages of nauplius VI to copepodid I. Maturation to adult was recorded when the metasomal T5 wings first appeared on females and with the development of a thick right antenna on the males.

### 2.4 Laboratory selection experiment to measure evolutionary response to crude oil toxicity

The selection experiment attempted to induce an evolutionary response to crude oil toxicity in the “pre‐oil spill” population, collected from the Gulf of Mexico region prior to the Deepwater Horizon oil spill (see section 2.1). Selection was imposed by transferring animals to increasingly higher oil concentrations (of the water‐accommodated fraction, WAF) across ~8 generations in June 2014. Approximately 500 animals from laboratory cultures were transferred to 50% WAF (diluted with 5 PSU water) and then maintained at that concentration for at least two generations. These cultures were subsequently transferred to 65% WAF (43 days, ~2 generations), then to 70% WAF (15 days, ~1 generation), and finally to 72.5% WAF (70 days, ~3 generations). Meanwhile, control cultures were cultured in 5 PSU water in the absence of crude oil. Both the “pre‐selection” (control) and “postselection” lines were cultured as described above and fed the cryptophyte alga *Rhodomonas salina*.

Selection for crude oil tolerance proved to be much more difficult to execute than prior selection experiments, such as selection in response to salinity (Lee et al., 2007, 2011). For instance, selection in response to low salinity is typically evident after only 2–6 generations (Lee et al., 2007). However, in this experiment more than three attempts were made to create crude oil selected lines, yet the lines frequently went extinct and had difficulty surviving for multiple generations. In addition, multiple replicate selection lines were started, but most went extinct.

Response to selection was quantified by comparing the “pre‐selection” and “post‐selection” lines in a common‐garden experiment. In order to remove the effects of environmental acclimation prior to the common‐garden experiment, both the “pre‐selection” and “post‐selection” lines were held at the same condition of 0% WAF prior to the experiment. The “pre‐selection” line was continuously reared at 0% WAF, whereas the “post‐selection” line was moved to 0% WAF for 30 days (~1.5 generations) prior to the experiment.

As in the previous experiment (previous section), eight ovigerous females were selected at random from each population and the full‐sib egg sacs, representing distinct genotypes, were excised from the female and split six ways across three treatments of WAF (this time, 0% [control], 72.5%, and 100% WAF), with two replicate vials per treatment. Between 8 to 10 eggs were split between two replicate vials per clutch for each oil concentration and population. With eight clutches per treatment, there were a total of 65–69 individual copepods per oil concentration for each population. Eggs were transferred to 20 ml scintillation vials containing ~10 ml of 5 PSU water. The vials were cultured in environmental chambers at 12°C, with a light cycle of 15L:9D, and fed the cryptophyte alga *Rhodomonas salina* in excess.

Survival and development time were measured in a similar manner as in the “pre‐” versus “post‐oil spill” common‐garden experiment above (previous section). In this experiment, observations of hatching, survival, and development time from hatching to metamorphosis were recorded daily. From metamorphosis to adult, survival and development time were recorded every other day, as in the previous experiment (previous section), and development time was averaged as the midpoint between observations. Animals were determined to reach the adult stage at the first appearance of the metasomal T5 wings in females and the development of a thick right antenna in males.

### 2.5 Statistical analyses

Survival data were analyzed in a linear mixed model framework using the glmer procedure, whereas development time data were analyzed using the lmer procedure in the lme4 package of R (Bates, Maechler, Bolker, & Walker, 2015; R Core Team 2016). Survival data were treated as binary and models included a logit link function. Effects of factors on survival and development time were examined (see next paragraph). Fixed effects (factors) included *Population*,* Oil Concentration*, and *Population* × *Oil Concentration*. Random effects included *Clutch* (genotype) and the random slope for *Oil Concentration* (with respect to *Clutch*). To determine the effects of each factor on the model, likelihood ratio tests were performed to compare goodness of fit between models with each factor included versus reduced models with the factor excluded.

Population comparisons were performed between “pre‐oil spill” versus “post‐oil spill” populations from the first experiment (section 2.3), and between “pre‐selection” versus “post‐selection” lines from the second experiment (section 2.4). We measured fitness‐related traits in terms of survival (hatch or not hatch, dead or alive from hatching to metamorphosis, dead or alive from metamorphosis to adult) and development time (in days, hatching to metamorphosis, metamorphosis to adult). We compared their traits at three crude oil concentrations (0% WAF control, 75% or 72.5% WAF, 100% WAF).

We also tested for the significance of differences in survival or development time between populations (“pre‐oil spill” vs. *“*post‐oil spill” and “pre‐selection” vs. “post‐selection”) at each oil concentration. These tests used the asymptotic normality of maximum likelihood estimates for differences in log odds, in the case of survival probabilities, and for differences in means, in the case of development time differences.

## 3 RESULTS

### 3.1 Comparisons between wild populations before and after the Deepwater Horizon oil spill

**Figure 1:**
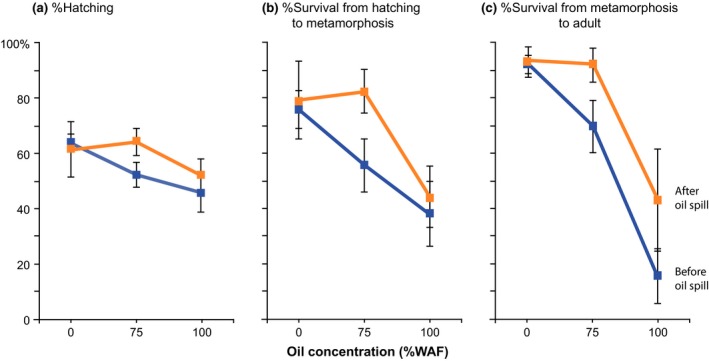
Hatching and survival across oil concentrations for populations of the copepod *Eurytemora affinis* collected before versus after the Deepwater Horizon oil spill of 2010 in the Gulf of Mexico. Graphs show (a) percentage hatching, (b) percentage survival from hatching to metamorphosis, and (c) percentage survival from metamorphosis to adult, for populations collected before the oil spill (blue, “pre‐oil spill”) versus after the oil spill (orange, “post‐oil spill”) observed across three oil concentrations (no oil control, 75% and 100% WAF, “water‐accommodated fraction”). Values shown are Mean ± *SE* for *N* = 8 clutches, with 10–12 eggs per clutch at each treatment. Values and significance in differences between the populations are listed in Table 3a–c

**Figure 2:**
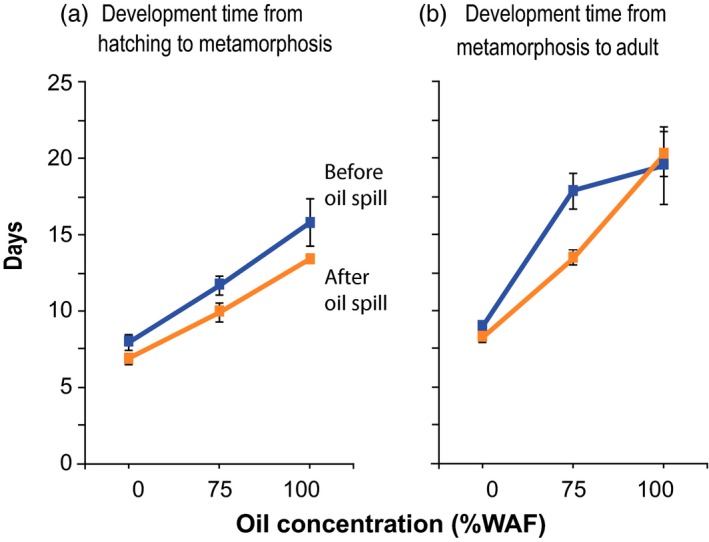
Development time across oil concentrations for populations of the copepod *E. affinis* collected before versus after the Deepwater Horizon oil spill of 2010 in the Gulf of Mexico. Graphs show development time (in days) from (a) hatching to metamorphosis and (b) metamorphosis to adult, for populations collected before the oil spill (blue, “pre‐oil spill”) versus after the oil spill (orange, “post‐oil spill”) across three oil concentrations (no oil control, 75% and 100% WAF, “water‐accommodated fraction”). Values shown are Mean ± *SE*. Values, sample size, and significance of differences between the populations are listed in Table 3d,e

**Figure 3:**
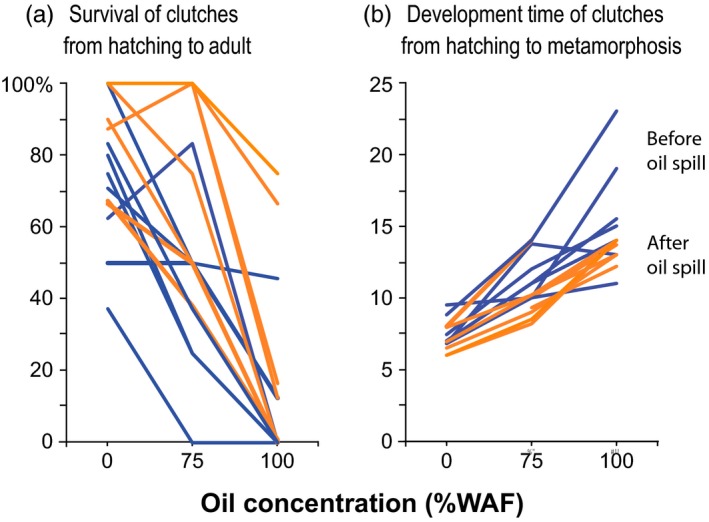
Survival and development time for individual clutches (representing genotypes) from populations of *E. affinis* collected before the Deepwater Horizon oil spill (blue, “pre‐oil spill”) versus after the oil spill (orange, “post‐oil spill”). Graphs show (a) percentage survival of clutches from hatching to adult for eight clutches and (b) development time (in days) of clutches from hatching to metamorphosis, across three oil concentrations (no oil control, 75% and 100% WAF, “water‐accommodated fraction”), with sample size (number of clutches) shown in Table 3d. Development time to metamorphosis (rather than to adult) is depicted here because many clutches did not survive to adulthood (Table 3e)

Our results revealed evolutionary shifts in fitness‐related traits in a population of the copepod *E. affinis* in response to crude oil toxicity, following the Deepwater Horizon oil spill of 2010 in the Gulf of Mexico (Figures 1, 2, 3; Tables 1, 2, 3). In the presence of crude oil, we found significantly greater survival and shorter development times in a population collected after the oil spill (“post‐oil spill”) relative to a population collected before the oil spill (“pre‐oil spill”) from same location (Blue Hammock Bayou, LA, USA; Figures 1, 2, Table 3). As our experiment was performed under common‐garden conditions, removing the effects of environmental acclimation, our results revealed rapid evolution of crude oil tolerance in a wild population at a particular location, directly following an oil spill.

**Table 1:** Effects of multiple factors on hatching and survival (using glmer in R)

	Hatching		Survival		Survival	
			Hatch to metamorphosis		Metamorphosis to adult	
	χ² (*df*)	*p* value	χ² (*df*)	*p* value	χ² (*df*)	*p* value
**(a) Before versus after the oil spill**						
*Fixed factors*						
Population (Before vs. after oil spill)	1.3 (1)	.25	9.1 (1)	**.0026**	8.1 (1)	**.0045**
Oil concentration	8.3 (2)	**.016**	43.1 (2)	**4.45e**−**10**	55.3 (2)	**9.78e**−**13**
Population × oil concentration	2.2 (2)	.33	3.5 (2)	.17	1.7 (2)	.42
*Random factors*						
*Clutch* (relative to model with fixed effects only)	0.16 (1)	.69	0.43 (1)	.51	7.3 (1)	**.0068**
Random slope for *oil concentration* (with respect to *Clutch*;* i.e., Clutch* × *Oil*)	6.6 (5)	.25	7.0 (5)	.22	3.3 (5)	.65
**(b) Before versus after laboratory selection**						
*Fixed factors*						
Population (before vs. after selection)	0.036 (1)	.84	3.0 (1)	.085	1.8 (1)	.18
Oil concentration	5.8 (2)	**.054**	23.5 (2)	**8.00e**−**06**	2.7 (2)	.26
Population × oil concentration	1.1 (2)	.59	11.6 (2)	**.0031**	3.2 (2)	.20
*Random factors*						
*Clutch* (relative to model with fixed effects only)	8.8 (1)	**.0030**	3.6 (1)	.058	0.0 (1)	1.00
Random slope for *oil concentration* (with respect to *Clutch*; i.e., *Clutch* × *Oil*)	0.37 (5)	.99	4.0 (5)	.55	3.8 (5)	.58

Results show effects of fixed factors of *Population*,* oil concentration*, and *Population* × *oil concentration*. Random effects include *Clutch* and random slope of *Oil Concentration (Clutch* × *Oil Concentration)*. Chi‐square (χ²) values, along with degrees of freedom (*df*), and *p‐*values are shown for likelihood ratio tests between full models versus models with each factor removed. Significant effects (*p *<* *.05) that improve the model are shown in bold.

**Table 2:** Effects of multiple factors on development time (using lmer in R)

	Development time		Development time	
	Hatching to metamorphosis		Metamorphosis to adult	
	χ² (*df*)	*p* value	χ² (*df*)	*p* value
**(a) Before versus after the oil spill**				
*Fixed factors*				
Population (before vs. after oil spill)	23.4 (1)	**1.30e**−**06**	15.0 (1)	**.00011**
Oil Concentration	191.9 (2)	**2.13e**−**42**	188.9 (1)	**9.52e**−**42**
Population × oil concentration	4.5 (2)	.11	20.8 (2)	**2.98e**−**05**
*Random Factors*				
*Clutch* (relative to model with fixed effects only)	15.8 (1)	**6.91e**−**05**	6.5 (1)	**.011**
Random slope for *oil concentration* (with respect to *Clutch*;* i.e., Clutch* × *Oil*)	11.5 (5)	**.0021**	157.6 (5)	**1.22e**−**31**
**(b) Before versus after laboratory selection**				
*Fixed Factors*				
Population (before vs. after selection)	3.3 (1)	.069	0.25 (1)	.62
Oil concentration	111.2 (2)	**7.16e**−**25**	20.4 (2)	**3.76e**−**05**
Population × Oil concentration	0.25 (2)	.88	11.3 (2)	**.0034**
*Random factors*				
*Clutch* (relative to model with fixed effects only)	24.1 (1)	**9.21e**−**07**	17.4 (1)	**2.95e**−**05**
Random Slope for *Oil Concentration* (with respect to *Clutch*;* i.e., Clutch* × *Oil*)	20.4 (5)	**9.53e**−**04**	0.22 (5)	0.99

Results show effects of fixed factors of *Population*,* Oil concentration*, and *Population* × *oil concentration*. Random effects include *Clutch* and random slope of *Oil Concentration (Clutch* × *Oil Concentration)*. Chi‐square (χ²) values, along with degrees of freedom (*df*), and *p*‐values are shown for likelihood ratio tests between full models versus models with each factor removed. Significant effects (*p *<* *.05) that improve the model are shown in bold.

**Table 3:** Survival and development time for Gulf of Mexico populations collected before versus after the Deepwater Horizon oil spill

	Before oil spill	After oil spill	*Z* statistic	*p*‐value
	Mean ± *SE* (*N*)	Mean ± *SE* (*N*)		
**(a) %Hatching**				
0% WAF	63.9 ± 3.2	61.5 ± 9.9	−0.34	.73
75% WAF	52.2 ± 4.5	64.2 ± 4.8	1.4	.15
100% WAF	45.6 ± 6.8	51.9 ± 6.1	0.67	.50
**(b) %Survival hatch to metamorphosis**				
0% WAF	75.8 ± 6.9	79.2 ± 14.0	0.59	.56
75% WAF	55.7 ± 9.5	82.3 ± 7.9	2.5	**.013**
100% WAF	38.0 ± 11.7	44.3 ± 11.2	0.86	.39
**(c) %Survival metamorphosis to adult**				
0% WAF	92.0 ± 3.4	92.8 ± 5.5	0.72	.47
75% WAF	69.6 ± 9.4	91.7 ± 6.3	2.3	**.023**
100% WAF	15.5 ± 10.0	42.9 ± 18.4	0.42	.67
**(d) Development time (days) from hatching to metamorphosis**				
0% WAF	7.98 ± 0.51 (8)	6.89 ± 0.36 (6)	−1.2	.22
75% WAF	11.68 ± 0.62 (7)	9.93 ± 0.64 (8)	−2.4	**.015**
100% WAF	15.79 ± 1.52 (7)	13.40 ± 0.27 (8)	−1.8	.065
**(e) Development time (days) from metamorphosis to adult**				
0% WAF	8.96 ± 0.49 (8)	8.24 ± 0.30 (6)	0.22	.82
75% WAF	17.86 ± 1.17 (7)	13.43 ± 0.38 (7)	3.5	**.00041**
100% WAF	19.50 ± 2.50 (2)	20.25 ± 1.49 (4)	0.49	.62

For percentage hatching and survival values (a, b, c), means among eight clutches are shown. Development time estimates (d, e) were often based on lower sample size due to mortality. The *Z* statistic is the difference in log odds of hatching or survival or the difference in mean development time between the populations (after oil spill – before oil spill) divided by the standard error of the difference in log odds. *p*‐values indicate significance of differences between the populations, with *p *<* *.05 shown in bold.

#### 3.1.1 Evolution of survival following the Deepwater Horizon oil spill

Our results revealed the evolution of increased survival of the “post‐oil spill” population, relative to the “pre‐oil spill” population, in the presence of the water‐accommodated fraction (WAF) of crude oil (Figure 1, Table 3b,c). This evolutionary shift following the oil spill was consistent with the significant effect of the factor *Population* on survival (Table 1a), both from hatching to metamorphosis (Figure 1b) and metamorphosis to adult (Figure 1c). The decline in hatching and survival at higher oil concentrations (Figure 1) was consistent with the significant effect of the factor *Oil Concentration* on these responses (Table 1a). The pre‐ and post‐oil spill populations did not appear to differ greatly in the pattern of their responses across oil concentrations (Figure 1), indicating little difference between populations in their plastic responses to oil concentration, consistent with the lack of significant *Population* × *Oil Concentration* interaction (Table 1a).

The post‐oil spill population exhibited significantly higher survival, from hatching to metamorphosis (Figure 1b) and from metamorphosis to adult (Figure 1c) at the intermediate (75% WAF) oil concentration (Table 3b,c), relative to the pre‐oil spill population. However, survival between the populations at the higher oil concentration (100% WAF) did not differ significantly (Figure 1b,c, Table 3b,c). While there was a trend toward higher survival at 100% WAF in the post‐oil spill population (Figure 1b,c), relative to the pre‐oil spill population, the variance was too high (Table 3b,c, standard error). Hatching rate was higher in the post‐ than pre‐oil spill population in the presence of oil (Figure 1a), but the differences were not significant (Table 3a). Unsurprisingly, and reassuringly, there were no significant differences in hatching or survival between the pre‐ and post‐oil spill populations at the control treatment (0% WAF), in the absence of WAF oil (Table 3a–c).

Given the evolutionary shifts in survival (Table 3b,c), one would expect a significant effect of clutch (genotype) on survival, as genetic variation in oil tolerance in the pre‐oil spill population would be required for natural selection to act and cause evolutionary change. We did find the effect of clutch to be significant for survival from metamorphosis to adult, but not for survival from hatching to metamorphosis (Table 1a, Figure 3a). Consistent with selection acting on survival, we found that genetic variation (variance among clutches) for survival at 75% WAF was reduced in the post‐oil spill population, relative to the pre‐oil spill population, for both survival from hatching to metamorphosis (Table 3b) and metamorphosis to adult (Table 3c).

#### 3.1.2 Evolution of development time following the Deepwater Horizon oil spill

The “post‐oil spill” population tended to exhibit more rapid development relative to the “pre‐oil spill” population in the presence of oil (Figure 2, Table 3d,e), indicating an evolutionary shift. This shift in development time following the oil spill was consistent with the significant effect of the factor *Population* on development time (Table 2a). Greatly retarded development at higher oil concentrations for both populations (Figure 2) was consistent with the highly significant effects of *Oil Concentration* on development time (Table 2a). The pre‐ and post‐oil spill populations exhibited divergent patterns of development time from metamorphosis to adult across oil concentrations (Figure 2b), revealing differences between populations in their plastic responses to oil concentration and consistent with the significant *Population* × *Oil Concentration* interaction (Table 2a).

Development time was significantly more rapid in the post‐oil spill population relative to the pre‐oil spill population at the intermediate oil concentration (75% WAF) (Table 3d,e), for both development time from hatching to metamorphosis (Figure 2a) and from metamorphosis to adult (Figure 2b). These significant differences at the intermediate 75% WAF oil concentration were consistent with the significant differences in survival at the intermediate oil concentration (previous section). There was a trend of faster development from hatching to metamorphosis at the high oil concentration (100% WAF) in the post‐oil spill population, relative to the pre‐oil spill population (Figure 2a), but the difference was not significant (Table 3d). For development time from metamorphosis to adult, there was no difference between the populations in at the 100% WAF oil concentration (Figure 2b, Table 3e). As expected, development time in the absence of oil (0% WAF control) showed no difference between the populations (Table 3d,e).

Consistent with the evolutionary shifts in development time (Figure 2, Table 3d,e), the effects of *Clutch* (genotype) on development time from hatching to metamorphosis and metamorphosis to adult were both significant (Table 2a). This result indicated the presence of ample genetic variation upon which natural selection could act (Figure 3b). The significant random slope for *Oil Concentration* (with respect to *Clutch*, or *Clutch* × *Oil Concentration*) (Table 2a) revealed the presence of genetic variation in response across oil concentrations (i.e., genetic variation in plasticity or reaction norms) within the populations (Figure 3b).

Also, consistent with the evolutionary shifts in development time, we found reduced genetic variation in development time in the post‐oil spill population relative to the pre‐oil spill population. We would expect a reduction in genetic variation for these traits if recent natural selection shaped the shifts in development time (Figure 2). Indeed, we did find a marked reduction in genetic variation in development time in the post‐oil spill population, as measured by variance among clutches (standard error), for development time from metamorphosis to adult at 75% WAF (Table 3e) and for development time from hatching to metamorphosis (Table 3d) and metamorphosis to adult (Table 3e) at 100% WAF.

### 3.2 Comparisons between populations before and after laboratory selection

**Figure 4:**
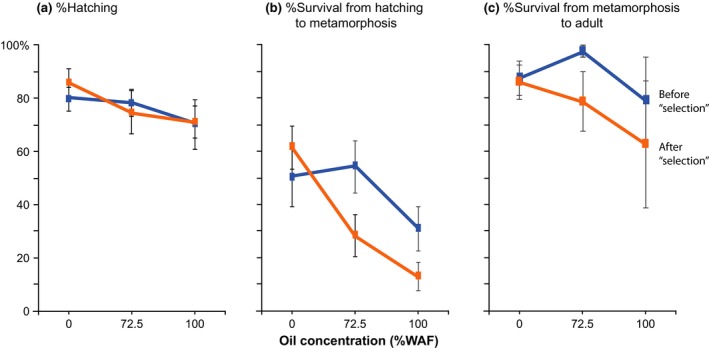
Hatching and survival across oil concentrations for the copepod *E. affinis* before versus after laboratory selection for crude oil tolerance (at 50%–72.5% WAF for 8 generations). The *E. affinis* population used in this experiment was collected from the Gulf of Mexico prior to the Deepwater Horizon oil spill of 2010. Graphs show (a) percentage hatching, (b) percentage survival from hatching to metamorphosis, and (c) percentage survival from metamorphosis to adult across three oil concentrations (no oil control, 72.5% and 100% WAF, “water‐accommodated fraction”), for copepod lines before (blue, “pre‐selection”) versus after (orange, “post‐selection”) laboratory selection. Values shown are Mean ± *SE* for eight clutches, with 6–10 eggs per clutch at each treatment. Values and significance of differences between the laboratory lines are listed in Table 4a–c

**Figure 5:**
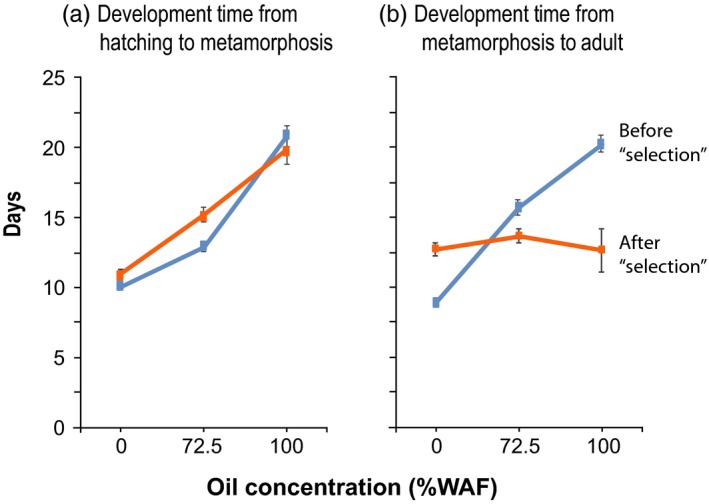
Development time across oil concentrations for the copepod *E. affinis* before versus after laboratory selection for crude oil tolerance (at 50%–72.5% WAF for 8 generations). The *E. affinis* population used in this experiment was collected from the Gulf of Mexico prior to the Deepwater Horizon oil spill of 2010. Graphs show development time (in days) from (a) hatching to metamorphosis and (b) metamorphosis to adult across three oil concentrations (no oil control, 72.5% and 100% WAF, “water‐accommodated fraction”), for copepod lines before (blue, “pre‐selection”) versus after (orange, “post‐selection”) laboratory selection. Values shown are Mean ± *SE*. Values, sample size, and significance of differences between the laboratory lines are listed in Table 4d,e

In contrast to the comparisons between the “pre‐” and “post‐oil spill” populations (Figure 1, Table 1A, 2A), patterns of evolution were less clear following laboratory selection for crude oil tolerance on a Gulf of Mexico (pre‐oil spill) population (Tables 1, 2, 4). We did not find greater survival in the “post‐selection” line (Figure 4), but did find a trend of shorter development time from metamorphosis to adult (Figure 5b, Table 4e). Our results suggest that adaptation in response to crude oil might be relatively difficult to achieve and might not proceed very rapidly, within the time frame of the selection experiment (~8 generations).

**Table 4:** Survival and development time before versus after laboratory selection for crude oil toxicity, using a Gulf of Mexico population collected prior to the Deepwater Horizon oil spill

	Before selection	After selection	*Z* statistic	*p*‐value
	Mean ± *SE* (*N*)	Mean ± *SE* (*N*)		
**(a) %Hatching**				
0% WAF	79.6 ± 4.5	85.6 ± 5.4	0.79	.43
72.5% WAF	77.9 ± 5.0	74.4 ± 8.2	−0.35	.72
100% WAF	70.1 ± 9.2	70.9 ± 6.0	−0.0026	1.0
**(b) %Survival hatch to metamorphosis**				
0% WAF	50.2 ± 10.8	61.5 ± 8.0	1.3	.191
72.5% WAF	54.2 ± 9.9	28.3 ± 8.0	1.8	.072
100% WAF	30.8 ± 8.2	13.0 ± 5.2	−2.1	**.033**
**(c) %Survival metamorphosis to adult**				
0% WAF	87.5 ± 6.7	85.9 ± 6.4	0.090	.93
72.5% WAF	97.6 ± 2.4	78.6 ± 11.2	−2.1	**.032**
100% WAF	79.2 ± 16.4	62.5 ± 23.9	−0.79	.43
**(d) Development time (days) from hatching to metamorphosis**				
0% WAF	7.98 ± 0.07 (8)	8.80 ± 0.19 (8)	0.49	.63
72.5% WAF	10.29 ± 0.28 (7)	12.15 ± 0.44 (7)	1.2	.24
100% WAF	16.61 ± 0.60 (6)	17.12 ± 0.99 (4)	0.49	.62
**(e) Development time (days) from metamorphosis to adult**				
0% WAF	10.60 ± 0.14 (8)	15.25 ± 0.52 (8)	2.5	**.013**
72.5% WAF	18.83 ± 0.66 (7)	16.39 ± 0.62 (7)	−1.4	.15
100% WAF	24.23 ± 0.73 (5)	15.17 ± 1.86 (3)	−1.5	.12

For percentage hatching and survival values (a, b, c), means among eight clutches are shown. Development time estimates (d, e) were often based on lower sample size due to mortality. The *Z* statistic is the difference in log odds of hatching or survival or the difference in mean development time between the lines (after selection – before selection) divided by the standard error. *p*‐values indicate significance of differences between the lines, with *p *<* *.05 shown in bold.

#### 3.2.1 Lack of evolution of survival following laboratory selection for crude oil tolerance

We observed no evidence for the evolution of increased tolerance of crude oil toxicity following ~8 generations of laboratory selection imposed on the pre‐oil spill population (Figure 4). In fact, the “postselection” line exhibited a trend toward reduced survival in response to crude oil toxicity relative to the “pre‐selection” line (Figure 4b,c, Table 4b,c). Overall, the pre and post‐selection lines did not differ significantly in their responses, as indicated by lack of effect of *Population* on hatching or survival (Table 1b, *p* > .05). The decline in hatching and survival with increasing oil concentration (Figure 4a,b) was consistent with the significant effect of *Oil Concentration* on these responses (Table 1b). The populations did differ in the pattern of survival from hatching to metamorphosis across oil concentrations (Figure 4b), consistent with the significant *Population* × *Oil Concentration* interaction (Table 1b).

Relative to the “pre‐selection” line, the “post‐selection” line exhibited significantly lower survival, in terms of survival from hatching to metamorphosis at the high oil concentration (100% WAF; Figure 4b, Table 4b) and survival from metamorphosis to adult at the intermediate oil concentration (75% WAF; Figure 4c, Table 4c). Overall survival from hatching to adult for the post‐selection line was quite low, of only 8.9% at the high (100% WAF) and 18.8% at the intermediate (72.5% WAF) oil concentrations, relative to 53.6% at the control with no oil (0% WAF). For hatching rate, we found no significant difference between the pre‐ and post‐oil spill populations across all oil concentrations (Figure 4a; Table 4a). Interestingly, once individuals managed to survive to metamorphosis, they tended to also survive to the adult stage. That is, while survival from hatching to metamorphosis in the presence of oil was generally quite low (13%–54%; Figure 4b, Table 4b), once animals reached metamorphosis, survival to adulthood was much higher (62%–98%; Figure 4b vs. c, Table 4b vs. c).

Given the lack of an evolutionary response in terms of survival, the lack of significant effect of *Clutch* (genotype) on survival was not surprising (Table 1b). On the other hand, there were significant effects of *Clutch* on hatching rate (Table 1b).

#### 3.2.2 Evolution of development time following laboratory selection for crude oil tolerance

Development from hatching to metamorphosis was retarded in the “post‐selection” line at the intermediate oil concentration (72.5% WAF; Figure 5a, Table 4d), but then accelerated from metamorphosis to adult at the intermediate and high oil concentrations (72.5% and 100% WAF; Figure 5b, Table 4e). While the factor *Population* did not show significant effects on development time (Table 2b) and pairwise differences between the pre and post‐selection lines were not significant due to low power resulting from high mortality (Table 4d,e), the patterns were nevertheless quite clear (Figure 5a,b). In fact, the pre and post‐selection lines exhibited highly divergent patterns in development time from metamorphosis to adult across oil concentrations (Figure 5b), reflected by the significant *Population* × *Oil Concentration* interaction (Table 2b). As in the previous experiment (Figure 2), development time was generally retarded at the higher oil concentrations for both pre and post‐selection lines, indicated by the significant effect of *Oil Concentration* on development time (Table 2b). However, the similar values for development times from metamorphosis to adult across oil concentrations in the post‐selection line (Figure 5b, orange line, Table 4e) deviated from this pattern.

The accelerated development of the post‐selection line from metamorphosis to adult (Figure 5b, orange line), relative to the pre‐selection line, was consistent with the accelerated development at 75% WAF observed in the post‐oil spill population (Figure 2b, orange line). But, the exceeding rapid development of the post‐selection line at 100% WAF (Figure 5b, orange line) was unlike the pattern seen for the post‐oil spill population (Figure 2b, orange line). This accelerated development in the post‐selection line was accompanied by exceedingly high mortality during development, especially from hatching to metamorphosis (see previous section; Figure 4b, Table 4b). In other words, most individuals died prior to metamorphosis in the post‐selection line in the presence of oil (Figure 4b, 13% survival at 100% WAF), but the individuals that did manage to survive to adulthood showed accelerated development relative to the pre‐selection line (Figure 5b).

The apparent evolutionary shifts in development time are consistent with the highly significant effects of *Clutch* (genotype) on development time (Table 2b). In addition, the significant random slope for *Oil Concentration* (with respect to *Clutch*, or *Clutch* × *Oil Concentration*; Table 2b) indicated the presence of genetic variation in plasticity for development time from hatching to metamorphosis (differences among clutches in tradeoffs at different oil concentrations). This significant effect of *Clutch* × *Oil Concentration* interaction was consistent with the shift in slope for development time following laboratory selection (Figure 5b, orange vs. blue lines).

## 4 DISCUSSION

### 4.1 Evolution of crude oil tolerance and life history in the wild

Our results revealed evidence for the evolution of crude oil tolerance in a population of the copepod *Eurytemora affinis* in the Gulf of Mexico following the Deepwater Horizon oil spill of 2010 (Figures 1 and 2, Tables 1a, 2a, and 3). We observed the evolution of fitness‐related traits, namely significantly higher survival and shorter development times in the “post‐oil spill” population, relative to the “pre‐oil spill” population, at the intermediate concentration of WAF crude oil (75% WAF, water‐accommodated fraction) (Table 3, Figures 1 and 2). Survival at this intermediate oil concentration in the post‐oil spill population was comparable to the high survival of both pre and post‐oil spill populations under the control conditions, containing no crude oil (0% WAF) (Table 3b,c, Figure 1b,c). Thus, the post‐oil spill population evolved full recovery of survival in the presence of 75% WAF oil, and with faster development rate relative to the pre‐oil spill population (Figure 2).

The high oil concentration (100% WAF) appeared to be very stressful and induced relatively high mortality for both pre‐ and post‐oil spill populations (Table 3b,c, Figure 1b,c). The post‐oil spill population did show a trend of higher survival than the pre‐oil spill population at the high oil concentration, but the difference was not significant (Table 3b,c). Additionally, development time at the high 100% WAF oil concentration was not significantly different between the populations (Table 3d,e). These results suggest that adaptation to the high oil concentration might be difficult to achieve. The high mortality suffered at 100% WAF in *E. affinis* was comparable to responses of other calanoid copepod species in response to WAF from the Deepwater Horizon Macondo Well crude oil (Almeda et al., 2013; Cohen et al., 2014). Although, these other studies examined acute or chronic responses of adults or nauplii to WAF, rather than survival during development, and did not investigate evolutionary responses.

Given the evolutionary shifts in survival and development time, one would expect a significant effect of *Clutch* (genotype) on these traits, as genetic variation in crude oil tolerance in the population would be required for natural selection to act and evolutionary change to occur. We did find the effect of *Clutch* to be significant for survival from metamorphosis to adult (Table 1a), but not for hatching to metamorphosis, likely due to insufficient power (Table 1a). For development time, the significant effect of *Clutch* for both the development to metamorphosis and adult stages (Tables 2a) likewise indicated the presence of genetic variation upon which selection could act. The significant effects of *Clutch* on survival and development time were consistent with the reduction in genetic variation for these traits in the post‐oil spill population, presumably due to selection acting on these traits (see Results). Directional selection acting on populations would reduce genetic variation at the relevant loci under selection.

The highly significant effects of *Clutch* × *Oil Concentration* (genotype × environment) interaction on development time (i.e., significant slope of *Oil Concentration* with respect to *Clutch*; Table 2a, Figure 3b) indicated the presence of genetic variation in plasticity, that is, genetic variation (clutch variation) in response across oil concentrations. This result indicated that selection could act on tradeoffs in development time across oil concentrations and that these tradeoffs (the mean slope of reaction norms) could evolve (Figure 2).

An intriguing question is how genetic variation in crude oil tolerance is maintained in the pre‐oil spill population (Figure 3), such that selection could act on this variation and enable survival and development time to evolve (Figures 1 and 2, Table 3). The location in the Gulf of Mexico that we sampled most intensely, Blue Hammock Bayou, Louisiana, was impacted by crude oil and the plankton community was adversely affected by the Deepwater Horizon oil spill (Sammarco et al., 2013) (see section 1). Perhaps the presence of natural oil seeps in the Gulf of Mexico and episodic injections of crude oil into the sediment and water (Anderson, Scalan, Parker, & Behrens, 1983; Stout & Payne, 2016; Stout, Payne, Ricker, Baker, & Lewis, 2016) had imposed temporally varying selection on the copepod population. Such conditions could have led to balancing selection acting to maintain genetic variation in crude oil tolerance in the copepod population, enhancing the evolutionary potential of the population when faced with changes in oil conditions (Posavi, Larget, Gelembiuk, & Lee, 2014; Turelli & Barton, 2004).

Additionally, the diapause egg bank could serve as a repository of genotypes from past selection regimes and could contribute to the maintenance of genetic variation in the populations (Ban & Minoda, 1992; Hairston, 1996; Posavi et al., 2014). That is, the presence of a diapause egg bank coupled with temporarily varying selection could extend the conditions under which genetic variation is maintained in the wild population (through balancing selection) (Posavi et al., 2014; Turelli & Barton, 2004). This diapause egg bank is replenished each year when the population goes into seasonal diapause (Ban, 1992), and contains eggs that could remain viable for ~10 years (Katajisto, 1996). We do not know the extent to which the diapause egg bank contributes to the effective population size (*N*_e_) of *E. affinis* populations, which is relatively large and estimated to be on the order of 10^6^–10^7^ (Winkler et al., 2008). The contribution of genetic diversity from the diapause egg bank is potentially large, given that the input of recruits from the egg bank can be considerable. For instance, ~50,000 *E. affinis* nauplii hatch from the egg bank per m^3^ per month in the Seine estuary (Glippa, Denis, Lesourd, & Souissi, 2014). Moreover, heritable phenotypic variation in the diapause egg bank has been found to differ from that of the water column population (Derry, Arnott, & Boag, 2010; Hairston, Kearns, & Ellner, 1996). Such genetic variation in the diapause egg bank could increase the evolutionary potential of *E. affinis* populations in response to a variety of stressors, including crude oil.

While we did observe clear heritable shifts in physiological tolerance (Figure 1) and performance (Figure 2) between the pre‐ and post‐oil spill populations, it is possible that factors other than evolutionary adaptation might have contributed to the differences. For instance, the pre‐oil population might have experienced genetic drift in the laboratory, resulting in some loss of crude oil tolerance and performance, causing a spurious divergence between the pre‐ and post‐oil spill populations. Additionally, relaxed selection for crude oil tolerance under laboratory conditions could have resulted in the loss of crude oil tolerance in the pre‐oil spill population cultured in the laboratory. Although, the fact that ample genetic variation for crude oil tolerance did persist in the laboratory population (Figure 3, blue lines) is counter to either genetic drift or relaxed selection removing most of the natural genetic variation from the population in the lab. The fact that the pre‐oil spill population did apparently disappear in the wild following the Deepwater Horizon oil spill, and only recovered more than a year later, suggests that an evolutionary response was likely important for its recovery.

### 4.2 Lower survival and rapid development following laboratory selection

The shifts in survival and development following laboratory selection (Figures 4, 5) differed markedly from the evolutionary changes experienced by the wild population following the oil spill (Figures 1, 2). Relative to the “pre‐selection” line, the lower survival in the “post‐selection” line (Table 4b,c, Figure 4b, 100% WAF; Figure 4c, 75% WAF) obviously did not reflect an adaptive evolutionary response. This lowered fitness in the post‐selection line might have resulted from the chronic cumulative stress of being reared in the presence of WAF oil for multiple generations, with insufficient time or genetic variation present in the laboratory line to enable selection to act to increase survival (see next section).

The increase in development time from hatching to metamorphosis at the intermediate oil concentration (72.5% WAF) in the post‐selection line differed markedly from the accelerated development of the post‐oil spill population (Figure 5a vs. 2a). This divergent pattern here might reflect the fact that there were only ~8 generations of selection in the laboratory, relative to 2–3 years (12–20 generations, with ~6 generations per year) of evolution in the wild. Perhaps selection for increased survival in crude oil over shorter periods of time led to tradeoffs resulting in slower development. Such a tradeoff between survival and development time is concordant with studies that show increased development time in response to selection for stress tolerance, similar to what has been observed for evolutionary responses to low salinity (Lee et al., 2003, 2007), as well as to other forms of stress, such as starvation and desiccation stress (Barrera & Medialdea, 1996; Chippindale et al., 1998; Harshman, Hoffmann, & Clark, 1999).

In contrast, the relatively few individuals that survived to metamorphosis in the post‐selection line (only 13%, Table 4b) tended to have rapid development from metamorphosis to adulthood (Figure 5b). This pattern was consistent with the pattern of evolution of the development in the wild population, where development became accelerated following the oil spill (Table 3, Figure 2). This decrease in development rate was conspicuous (Figure 5b), even though the mean differences were not significant due to the small sample size arising from exceedingly high mortality after selection (Figure 4b, Table 4b, N = 5 and *N* = 3). The effect of *Clutch* (genotype) was highly significant for development time (Table 2b), indicating the presence of genetic variation upon which natural selection could act.

Most notably, there was a conspicuous shift in how the pre and post‐selection lines responded across oil concentrations, in terms of development time from metamorphosis to adult, as indicated by the shift in slope of reaction norms between the lines (Figure 5b). The shift in slope between the pre and post‐selection lines was consistent with the significant effect of *Clutch* × *Oil Concentration* (genotype × environment) interaction on development during the prior life history stage, from hatching to metamorphosis (Table 2b). This significant interaction indicated the presence of genetic variation in reaction norms, or tradeoffs among clutches in development time across oil concentrations, such that clutches varied in their performance at high versus low oil concentrations. Selection on these tradeoffs across oil concentrations would lead to evolutionary shifts in reaction norms and changes in slope, as we observed following laboratory selection (Figure 5b). The effect of *Clutch* × *Oil Concentration* interaction on development time was not significant at the following life history stage (from metamorphosis to adult, Table 2b), likely due to low power resulting from the small numbers of survivors (Table 4).

For laboratory selection, the period from hatching to metamorphosis appeared to be the critical life history stage, where survival was the lowest (Figure 4b, Table 4b). This was far more true in the laboratory selection lines than in the wild populations (Figure 1b, Table 3b). Interestingly, we found that once individuals survived to metamorphosis during laboratory selection, they tended to also survive to the adult stage (Table 4b vs. c). The high mortality from hatching to metamorphosis (Figure 4b, Table 4b) suggests that selection acts most intensively during this life history stage. This pattern is similar to the case for selection on low salinity tolerance in *E. affinis* (Lee et al., 2003, 2007), where selection acts predominantly on the life history stage prior to metamorphosis. Selection acting more intensely during the early life‐history stages is commonly found in many different animal species (Garrido et al., 2015; Plough, Shin, & Hedgecock, 2016; Prasad, Shakarad, Anitha, Rajamani, & Joshi, 2001).

### 4.3 Rapid evolution of crude oil tolerance in the wild and the challenges of inducing an evolutionary response in the lab

This study provided novel insights into the speed and process of evolutionary adaptation during recovery from a catastrophic oil spill. In particular, this study provided a rare examination of the evolution of tolerance and performance of an animal population in response to crude oil, by directly comparing populations at a given location before versus after an oil spill. Given the relatively rapid recovery of the population of *E. affinis* in the Gulf of Mexico following the Deepwater Horizon 2010 oil spill (2–3 years, ~12–20 generations), we had expected that crude oil tolerance would be relatively easy to induce by imposing selection in the laboratory. On the contrary, we found that ~8 generations of laboratory selection (Figures 4, 5) was insufficient to replicate the evolutionary patterns found in the wild population (Figures 1, 2, 3). Namely, we did not find evolution of increased survival in the post‐selection line (Figure 4, Table 4b,c). Although, given that significant evolutionary shifts did occur in development time (Figure 5; significant *Population* × *Oil*, Table 2b), it is possible that the post‐selection line was in the process of responding to selection, but required longer periods of time for selection to increase survival in crude oil.

Alternatively, the difficulties of inducing a selection response for crude oil tolerance in the laboratory might have stemmed from insufficient standing genetic variation for the relevant traits in the laboratory. Although, the subsampling and measurement of clutch traits from the pre‐oil spill population did reveal a considerable amount of genetic variation in crude oil tolerance, upon which selection could act (Figure 3a). It is possible that even with the presence of significant genetic variation for crude oil tolerance in the pre‐selection line (=pre‐oil spill population, Figure 3, blue lines), the starting population size might have still been insufficiently large to capture the relevant alleles necessary for a selection response to crude oil in the laboratory. Unlike the laboratory selection lines, wild *E. affinis* populations receive recruits from a diapause egg bank, which is likely to harbor additional genetic diversity (see previous section) (Derry et al., 2010; Glippa et al., 2014; Hairston et al., 1996).

Our results suggest that recovery from an oil spill would be difficult and slow in absolute terms, even for a copepod that has short generation time (~15–20 days). We found that executing laboratory selection in response to crude oil was comparatively more difficult relative to the ease to which laboratory populations of *E. affinis* could respond to selection in response to salinity or temperature stress (Ketzner & Bradley, 1982; Lee et al., 2007, 2011). The selection experiments in this study used similar approaches as prior studies, with similar starting population size (hundreds of copepods). Difficulties in inducing an evolutionary response to crude oil was also found in populations of Trinidad guppies, where populations from oil‐polluted tributaries showed little evidence of local adaptation in survival and growth (Rolshausen et al., 2015).

Perhaps selection on crude oil tolerance is more difficult to perform because of greater numbers of targets of selection than other stressors (such as salinity or temperature), given the large number of chemicals contained within crude oil (Almeda et al., 2013; Cohen et al., 2014). The copepod *E. affinis* does possess the cytochrome P450 of the CYP3027 family (Posavi, 2015), found to show increased expression in response to crude oil in other copepods (Han et al., 2014, 2015). It would be worth exploring the specific stressors contained within the complex cocktail of crude oil and the specific targets of selection required for crude oil tolerance to evolve, such as specific CYP detoxification enzymes and potentially genes involved in the aryl hydro‐carbon receptor (AHR) signaling pathway (Reid et al., 2016; Whitehead, Pilcher, et al. 2012). Much work remains to be accomplished to dissect the specific physiological mechanisms involved in crude oil tolerance and how these mechanisms could rapidly evolve in order to survive catastrophic oil spills.

## DATA ARCHIVING STATEMENT

Data for this study are available at: Data available from the Dryad Digital Repository: https://doi.org/10.5061/dryad.t6d23.

## References

[eva12502-bib-0001] Almeda, R. , Wambaugh, Z. , Wang, Z. , Hyatt, C. , Liu, Z. , & Buskey, E. J. (2013). Interactions between zooplankton and crude oil: Toxic effects and bioaccumulation of polycyclic aromatic hydrocarbons. PLoS ONE, 8, e67212. 23840628 10.1371/journal.pone.0067212 PMC3696092

[eva12502-bib-0002] Anderson, R. K. , Scalan, R. S. , Parker, P. L. , & Behrens, E. W. (1983). Seep oil and gas in Gulf of Mexico slope sediment. Science, 222, 619–621. 17843839 10.1126/science.222.4624.619

[eva12502-bib-0003] Ban, S. (1992). Effects of photoperiod, temperature, and population density on induction of diapause egg production in *Eurytemora affinis* (Copepoda: Calanoida) in Lake Ohnuma, Hokkaido, Japan. Journal of Crustacean Biology, 12, 361–367.

[eva12502-bib-0004] Ban, S. , & Minoda, T. (1992). Hatching of diapause eggs of *Eurytemora affinis* (Copepoda: Calanoida) collected from lake‐bottom sediments. Journal of Crustacean Biology, 12, 51–56.

[eva12502-bib-0005] Barata, C. , Calbet, A. , Saiz, E. , Ortiz, L. , & Bayona, J. M. (2005). Predicting single and mixture toxicity of petrogenic polycyclic aromatic hydrocarbons to the copepod *Oithona davisae* . Environmental Toxicology and Chemistry, 24, 2992–2999. 16398138 10.1897/05-189r.1

[eva12502-bib-0006] Barrera, R. , & Medialdea, V. (1996). Development time and resistance to starvation of mosquito larvae. Journal of Natural History, 30, 447–458.

[eva12502-bib-0007] Bates, D. , Maechler, M. , Bolker, B. , & Walker, S. (2015). Fitting linear mixed‐effects models using lme4. Journal of Statistical Software, 67, 1–48.

[eva12502-bib-0008] Bejarano, A. C. , Chandler, G. T. , He, L. , & Coull, B. C. (2006). Individual to population level effects of South Louisiana crude oil water accommodated hydrocarbon fraction (WAF) on a marine meiobenthic copepod. Journal of Experimental Marine Biology and Ecology, 332, 49–59.

[eva12502-bib-0009] Berdugo, V. , Harris, R. P. , & O'Hara, S. C. (1977). The effect of petroleum hydrocarbons on reproduction of an estuarine planktonic copepod in laboratory cultures. Marine Pollution Bulletin, 8, 138–143.

[eva12502-bib-0010] Carrera‐Martinez, D. , Mateos‐Sanz, A. , Lopez‐Rodas, V. , & Costas, E. (2011). Adaptation of microalgae to a gradient of continuous petroleum contamination. Aquatic Toxicology, 101, 342–350. 21216344 10.1016/j.aquatox.2010.11.009

[eva12502-bib-0011] Chippindale, A. K. , Gibbs, A. G. , Sheik, M. , Yee, K. J. , Djawdan, M. , Bradley, T. J. , & Rose, M. R. (1998). Resource acquisition and the evolution of stress resistance in *Drosophila melanogaster* . Evolution, 52, 1342–1352. 28565385 10.1111/j.1558-5646.1998.tb02016.x

[eva12502-bib-0012] Cohen, J. H. , McCormick, L. R. , & Burkhardt, S. M. (2014). Effects of dispersant and oil on survival and swimming activity in a marine copepod. Bulletin of Environmental Contamination and Toxicology, 92, 381–387. 24402000 10.1007/s00128-013-1191-4

[eva12502-bib-0013] Derry, A. M. , Arnott, S. E. , & Boag, P. T. (2010). Evolutionary shifts in copepod acid tolerance in an acid‐recovering lake indicated by resurrected resting eggs. Evolutionary Ecology, 24, 133–145.

[eva12502-bib-0014] Faksness, L.‐G. , Brandvik, P. J. , & Sydnes, L. K. (2008). Composition of the water accommodated fractions as a function of exposure times and temperatures. Marine Pollution Bulletin, 56, 1746–1754. 18715599 10.1016/j.marpolbul.2008.07.001

[eva12502-bib-0015] Forth, H. , Mitchelmore, C. L. , Morris, J. M. , & Lipton, J. (2017). Characterization of oil and water accommodated fractions used to conduct aquatic toxicity testing in support of the Deepwater Horizon oil spill natural resource damage assessment. Environmental Toxicology and Chemistry, 36, 1450–1459. 27805278 10.1002/etc.3672

[eva12502-bib-0016] Garrido, S. , Ben‐Hamadou, R. , Santos, A. M. P. , Ferreira, S. , Teodósio, M. A. , Cotano, U. , … Ré, P. (2015). Born small, die young: Intrinsic, size‐selective mortality in marine larval fish. Scientific Reports, 5, 17065. 26597385 10.1038/srep17065 PMC4657020

[eva12502-bib-0017] Girling, A. E. (1989). Preparation of aqueous media for aquatic toxicity testing of oils and oil‐based products: A review of the published literature. Chemosphere, 19, 1635–1641.

[eva12502-bib-0018] Glippa, O. , Denis, L. , Lesourd, S. , & Souissi, S. (2014). Seasonal fluctuations of the copepod resting egg bank in the middle Seine estuary, France: Impact on the nauplii recruitment. Estuarine and Coastal Marine Science, 142, 60–67.

[eva12502-bib-0019] Graham, W. M. , Condon, R. H. , Carmichael, R. H. , D'Ambra, I. , Patterson, H. K. , Linn, L. J. , & Hernandez, F. J. Jr (2010). Oil carbon entered the coastal planktonic food web during the Deepwater Horizon oil spill. Environmental Research Letters, 5, 045301.

[eva12502-bib-0020] Griffiths, S. K. (2012). Oil release from Macondo well MC252 following the Deepwater Horizon accident. Environmental Science & Technology, 46, 5616–5622. 22506853 10.1021/es204569t

[eva12502-bib-0021] Hairston, N. G. (1996). Zooplankton egg banks as biotic reservoirs in changing environments. Limnology and Oceanography, 41, 1087–1092.

[eva12502-bib-0022] Hairston, N. G. , Kearns, C. M. , & Ellner, S. P. (1996). Phenotypic variation in a zooplankton egg bank. Ecology, 77, 2382–2392.

[eva12502-bib-0023] Han, J. , Won, E.‐J. , Hwang, D.‐S. , Shin, K.‐H. , Lee, Y. S. , Leung, K. M.‐Y. , … Lee, J.‐S. (2014). Crude oil exposure results in oxidative stress‐mediated dysfunctional development and reproduction in the copepod *Tigriopus japonicus* and modulates expression of cytochrome P450 (CYP) genes. Aquatic Toxicology, 152, 308–317. 24813263 10.1016/j.aquatox.2014.04.027

[eva12502-bib-0024] Han, J. , Won, E.‐J. , Kim, H.‐S. , Nelson, D. R. , Lee, S.‐J. , Park, H. G. , & Lee, J.‐S. (2015). Identification of the full 46 cytochrome P450 (CYP) complement and modulation of CYP expression in response to water‐accommodated fractions of crude oil in the cyclopoid copepod *Paracyclopina nana* . Environmental Science & Technology, 49, 6982–6992. 25942333 10.1021/acs.est.5b01244

[eva12502-bib-0025] Harshman, L. G. , Hoffmann, A. A. , & Clark, A. G. (1999). Selection for starvation resistance in *Drosophila melanogaster*: physiological correlates, enzyme activities and multiple stress responses. Journal of Evolutionary Biology, 12, 370–379.

[eva12502-bib-0026] Katajisto, T. (1996). Copepod eggs survive a decade in the sediments of the Baltic Sea. Hydrobiologia, 320, 153–159.

[eva12502-bib-0027] Ketzner, P. A. , & Bradley, B. P. (1982). Rate of environmental change and adaptation in the copepod *Eurytemora affinis* . Evolution, 36, 298–306. 28563172 10.1111/j.1558-5646.1982.tb05045.x

[eva12502-bib-0028] Kimmel, D. G. , Miller, W. D. , & Roman, M. R. (2006). Regional scale climate forcing of mesozooplankton dynamics in Chesapeake Bay. Estuaries and Coasts, 29, 375–387.

[eva12502-bib-0029] Lee, C. E. (1999). Rapid and repeated invasions of fresh water by the saltwater copepod *Eurytemora affinis* . Evolution, 53, 1423–1434. 28565555 10.1111/j.1558-5646.1999.tb05407.x

[eva12502-bib-0030] Lee, C. E. (2000). Global phylogeography of a cryptic copepod species complex and reproductive isolation between genetically proximate “populations”. Evolution, 54, 2014–2027. 11209778 10.1111/j.0014-3820.2000.tb01245.x

[eva12502-bib-0032] Lee, C. E. (2016). Evolutionary mechanisms of habitat invasions, using the copepod *Eurytemora affinis* as a model system. Evolutionary Applications, 9, 248–270. 27087851 10.1111/eva.12334 PMC4780390

[eva12502-bib-0033] Lee, C. E. , Kiergaard, M. , Gelembiuk, G. W. , Eads, B. D. , & Posavi, M. (2011). Pumping ions: Rapid parallel evolution of ionic regulation following habitat invasions. Evolution, 65, 2229–2244. 21790571 10.1111/j.1558-5646.2011.01308.x

[eva12502-bib-0034] Lee, C. E. , Moss, W. E. , Olson, N. , Chau, K. F. , Chang, Y.‐M. , & Johnson, K. E. (2013). Feasting in fresh water: Impacts of food concentration on freshwater tolerance and the evolution of food × salinity response during the expansion from saline into freshwater habitats. Evolutionary Applications, 6, 673–689. 23789033 10.1111/eva.12054 PMC3684747

[eva12502-bib-0035] Lee, C. E. , & Petersen, C. H. (2003). Effects of developmental acclimation on adult salinity tolerance in the freshwater‐invading copepod *Eurytemora affinis* . Physiological and Biochemical Zoology, 76, 296–301. 12905115 10.1086/375433

[eva12502-bib-0036] Lee, C. E. , Remfert, J. L. , & Chang, Y.‐M. (2007). Response to selection and evolvability of invasive populations. Genetica, 129, 179–192. 16915512 10.1007/s10709-006-9013-9

[eva12502-bib-0037] Lee, C. E. , Remfert, J. L. , & Gelembiuk, G. W. (2003). Evolution of physiological tolerance and performance during freshwater invasions. Integrative and Comparative Biology, 43, 439–449. 21680452 10.1093/icb/43.3.439

[eva12502-bib-0038] Liu, Y. , & Kujawinski, E. B. (2015). Chemical composition and potential environmental impacts of water‐soluble polar crude oil components inferred from ESI FT‐ICR MS. PLoS ONE, 10, e0136376. 26327219 10.1371/journal.pone.0136376 PMC4556654

[eva12502-bib-0039] Livdāne, L. , Putnis, I. , Rubene, G. , Elferts, D. , & Ikauniece, A. (2016). Baltic herring prey selectively on older copepodites of *Eurytemora affinis* and *Limnocalanus macrurus* in the Gulf of Riga. Oceanologia, 58, 46–53.

[eva12502-bib-0040] Melbye, A. G. , Brakstad, O. G. , Hokstad, J. N. , Gregersen, I. K. , Hansen, B. H. , Booth, A. M. , … Tollefsen, K. E. (2009). Chemical and toxicological characterization of an unresolved complex mixture‐rich biodegraded crude oil. Environmental Toxicology and Chemistry, 28, 1815–1824. 19413365 10.1897/08-545.1

[eva12502-bib-0041] Montagna, P. A. , Baguley, J. G. , Cooksey, C. , & Hyland, J. L. (2017). Persistent impacts to the deep soft‐bottom benthos one year after the Deepwater Horizon event. Integrated Environmental Assessment and Management, 13, 342–351. 27144656 10.1002/ieam.1791

[eva12502-bib-0042] Neff, J. M. (2002). Bioaccumulation in marine organisms: Effect of contaminants from oil well produced water. Oxford: Elsevier.

[eva12502-bib-0043] Nevo, E. , & Lavie, B. (1989). Selection of allozyme genotypes of two species of marine gastropods (genus *Littorina*) in experiments of environmental stress by nonionic detergent and crude oil‐surfactant mixtures. Genetics Selection Evolution, 21, 295–302.

[eva12502-bib-0044] Nie, Y. , Chi, C.‐Q. , Fang, H. , Liang, J.‐L. , Lu, S.‐L. , Lai, G.‐L. , … Wu, X.‐L. (2014). Diverse alkane hydroxylase genes in microorganisms and environments. Scientific Reports, 4, 4968. 24829093 10.1038/srep04968 PMC4021335

[eva12502-bib-0045] Ott, F. S. , Harris, R. P. , & O'Hara, S. C. M. (1978). Acute and sublethal toxicity of naphthalene and three methylated derivatives to the estuarine copepod, *Eurytemora affinis* . Marine Environmental Research, 1, 49–58.

[eva12502-bib-0046] Pendergraft, M. A. , & Rosenheim, B. E. (2014). Varying relative degradation rates of oil in different forms and environments revealed by ramped pyrolysis. Environmental Science & Technology, 48, 10966–10974. 25105342 10.1021/es501354c

[eva12502-bib-0047] Plough, L. V. , Shin, G. , & Hedgecock, D. (2016). Genetic inviability is a major driver of type III survivorship in experimental families of a highly fecund marine bivalve. Molecular Ecology, 25, 895–910. 26756438 10.1111/mec.13524

[eva12502-bib-0048] Posavi, M. , Larget, B. , Gelembiuk, G. W. , & Lee, C. E. (2014). Testing for beneficial reversal of dominance during salinity shifts in the invasive copepod *Eurytemora affinis*, and implications for the maintenance of genetic variation. Evolution, 68, 3166–3183. 25135455 10.1111/evo.12502

[eva12502-bib-0049] Posavi, M. (2015). *Evolutionary mechanisms of rapid adaptation during freshwater invasions by the saline copepod Eurytemora affinis* Ph.D. Thesis, Department of Zoology, University of Wisconsin.

[eva12502-bib-0050] Prasad, N. G. , Shakarad, M. , Anitha, D. , Rajamani, M. , & Joshi, A. (2001). Correlated responses to selection for faster development and early reproduction in *Drosophila*: The evolution of larval traits. Evolution, 55, 1363–1372. 11525460 10.1111/j.0014-3820.2001.tb00658.x

[eva12502-bib-0051] R Core Team (2016). R: A language and environment for statistical computing. Vienna, Austria: R Foundation for Statistical Computing.

[eva12502-bib-0052] Reid, N. M. , Proestou, D. A. , Clark, B. W. , Warren, W. C. , Colbourne, J. K. , Shaw, J. R. , … Whitehead, A. (2016). The genomic landscape of rapid repeated evolutionary adaptation to toxic pollution in wild fish. Science, 354, 1305–1308. 27940876 10.1126/science.aah4993 PMC5206662

[eva12502-bib-0053] Robson, W. J. , Sutton, P. A. , McCormack, P. , Chilcott, N. P. , & Rowland, S. J. (2017). Class type separation of the polar and apolar components of petroleum. Analytical Chemistry, 89, 2919–2927. 28194945 10.1021/acs.analchem.6b04202

[eva12502-bib-0054] Rolshausen, G. , Phillip, D. A. T. , Beckles, D. M. , Akbari, A. , Ghoshal, S. , Hamilton, P. B. , … Hendry, A. P. (2015). Do stressful conditions make adaptation difficult? Guppies in the oil‐polluted environments of southern Trinidad. Evolutionary Applications, 8, 854–870. 26495039 10.1111/eva.12289 PMC4610383

[eva12502-bib-0055] Romero‐Lopez, J. , Lopez‐Rodas, V. , & Costas, E. (2012). Estimating the capability of microalgae to physiological acclimatization and genetic adaptation to petroleum and diesel oil contamination. Aquatic Toxicology, 124–125, 227–237. 10.1016/j.aquatox.2012.08.001 22982500

[eva12502-bib-0056] Saiz, E. , Movilla, J. , Yebra, L. , Barata, C. , & Calbet, A. (2009). Lethal and sublethal effects of naphthalene and 1,2‐dimethylnaphthalene on naupliar and adult stages of the marine cyclopoid copepod *Oithona davisae* . Environmental Pollution, 157, 1219–1226. 19147262 10.1016/j.envpol.2008.12.011

[eva12502-bib-0057] Sammarco, P. W. , Kolian, S. R. , Warby, R. A. F. , Bouldin, J. L. , Subra, W. A. , & Porter, S. A. (2013). Distribution and concentrations of petroleum hydrocarbons associated with the BP/Deepwater Horizon Oil Spill, Gulf of Mexico. Marine Pollution Bulletin, 73, 129–143. 23831318 10.1016/j.marpolbul.2013.05.029

[eva12502-bib-0058] Shaheen, P. A. , Stehlik, L. L. , Meise, C. J. , Stoner, A. W. , Manderson, J. P. , & Adams, D. L. (2001). Feeding behavior of newly settled winter flounder (*Pseudopleuronectes americanus*) on calanoid copepods. Journal of Experimental Marine Biology and Ecology, 257, 37–51. 11165298 10.1016/s0022-0981(00)00335-x

[eva12502-bib-0059] Singer, M. M. , Aurand, D. V. , Coelho, G. M. , Bragin, G. E. , Clark, J. R. , Sowby, M. , & Tjeerdema, R. S. (2001). Making, measuring, and using water‐accommodated fractions of petroleum for toxicity testing. International Oil Spill Conference Proceedings, 2001, 1269–1274.

[eva12502-bib-0060] Stout, S. A. , & Payne, J. R. (2016). Macondo oil in deep‐sea sediments: Part 1—sub‐sea weathering of oil deposited on the seafloor. Marine Pollution Bulletin, 111, 365–380. 27488960 10.1016/j.marpolbul.2016.07.036

[eva12502-bib-0061] Stout, S. A. , Payne, J. R. , Ricker, R. W. , Baker, G. , & Lewis, C. (2016). Macondo oil in deep‐sea sediments: Part 2—Distribution and distinction from background and natural oil seeps. Marine Pollution Bulletin, 111, 381–401. 27509822 10.1016/j.marpolbul.2016.07.041

[eva12502-bib-0062] Tsvetnenko, Y. , & Evans, L. (2002). Improved approaches to ecotoxicity testing of petroleum products. Marine Pollution Bulletin, 45, 148–156. 12398379 10.1016/s0025-326x(02)00124-8

[eva12502-bib-0063] Turelli, M. , & Barton, N. H. (2004). Polygenic variation maintained by balancing selection: Pleiotropy, sex‐dependent allelic effects and GxE interactions. Genetics, 166, 1053–1079. 15020487 10.1093/genetics/166.2.1053 PMC1470722

[eva12502-bib-0064] Vecchione, M. (1989). Zooplankton distribution in three estuarine bayous with different types of anthropogenic influence. Estuaries, 12, 169–179.

[eva12502-bib-0065] Viitasalo, M. , Flinkman, J. , & Viherluoto, M. (2001). Zooplanktivory in the Baltic Sea: A comparison of prey selectivity by *Clupea harengus* and *Mysis mixta*, with reference to prey escape reactions. Marine Ecology Progress Series, 216, 191–200.

[eva12502-bib-0066] Whitehead, A. , Dubansky, B. , Bodinier, C. , Garcia, T. I. , Miles, S. , Pilley, C. , … Galvez, F. (2012). Genomic and physiological footprint of the Deepwater Horizon oil spill on resident marsh fishes. Proceeding of the National Academy of Sciences USA, 109, 20298–20302. 10.1073/pnas.1109545108 PMC3528528 21949382

[eva12502-bib-0067] Whitehead, A. , Pilcher, W. , Champlin, D. , & Nacci, D. (2012). Common mechanism underlies repeated evolution of extreme pollution tolerance. Proceedings of the Royal Society B: Biological Science, 279, 427–433. 10.1098/rspb.2011.0847 PMC3234547 21733895

[eva12502-bib-0068] Winkler, G. , Dodson, J. J. , Bertrand, N. , Thivierge, D. , & Vincent, W. F. (2003). Trophic coupling across the St. Lawrence River estuarine transition zone. Marine Ecology Progress Series, 251, 59–73.

[eva12502-bib-0069] Winkler, G. , Dodson, J. J. , & Lee, C. E. (2008). Heterogeneity within the native range: Population genetic analyses of sympatric invasive and noninvasive clades of the freshwater invading copepod *Eurytemora affinis* . Molecular Ecology, 17, 415–430. 17868296 10.1111/j.1365-294X.2007.03480.x

[eva12502-bib-0070] Yan, B. , Passow, U. , Chanton, J. P. , Nöthig, E.‐M. , Asper, V. , Sweet, J. , … Pak, D. (2016). Sustained deposition of contaminants from the Deepwater Horizon spill. Proceeding of the National Academy of Sciences USA, 113, E3332–E3340. 10.1073/pnas.1513156113 PMC4914201 27247393

